# Current Perspectives in Human T-Cell Leukemia Virus Type 1 Infection and Its Associated Diseases

**DOI:** 10.3389/fmed.2022.867478

**Published:** 2022-04-08

**Authors:** Michi Miura, Tadasuke Naito, Mineki Saito

**Affiliations:** Department of Microbiology, Kawasaki Medical School, Kurashiki, Japan

**Keywords:** HTLV-1, ATL, HAM/TSP, tax, HBZ

## Abstract

Human T-cell leukemia virus type 1 (HTLV-1) is a replication-competent human retrovirus associated with two distinct types of diseases: a malignancy of mature CD4^+^ T cells called adult T-cell leukemia-lymphoma (ATL) and a chronic inflammatory central nervous system disease HTLV-1-associated myelopathy/tropical spastic paraparesis (HAM/TSP). It was the first human retrovirus ever associated with a human cancer. Although most HTLV-1-infected individuals remain asymptomatic for life, a subpopulation develops ATL or HAM/TSP. Although the factors that cause these different manifestations of HTLV-1 infection are not fully understood, accumulating evidence suggests that the complex virus-host interactions, as well as the host immune response against HTLV-1 infection, appear to regulate the development of HTLV-1-associated diseases. This review outlines and discusses the current understanding, ongoing developments, and future perspectives of HTLV-1 research.

## Introduction

Human T-cell leukemia virus type 1 (HTLV-1) belongs to the genus Deltaretrovirus of the Orthoretrovirinae subfamily and infects approximately 5–10 million individuals worldwide (1). HTLV-1 is a causative agent of adult T-cell leukemia (ATL), an aggressive form of T-cell malignancy. Some 4–5% of HTLV-1 carriers develop ATL during their lifetime (2). HTLV-1 also causes HTLV-1-associated myelopathy/tropical spastic paraparesis (HAM/TSP) in another 0.25–4% of infections (3). In HAM/TSP, the corticospinal (pyramidal) tracts of the spinal cord are severely affected by inflammatory reactions. This inflammatory disease results in irreversible paraparesis of the lower limbs (4). The majority of infected individuals remain lifelong asymptomatic carriers (ACs).

Since the discovery of HTLV-1 and its association with these diseases in the 1980s (5–7), significant progress has been made in molecular studies of this virus and the infected host, from sequencing the viral genome to revealing the mechanisms of viral gene regulation and from identifying molecular markers to developing molecular therapeutics to treat the disease. However, HTLV-1 infection remains a threat to the human population. Although some new treatments have been developed, the prognosis of ATL is poor (8), and HTLV-1 significantly deteriorates the quality of life of HAM/TSP patients (9).

HTLV-1 is a latent virus. The host immune system is unable to clear the virus; therefore, HTLV-1 persists in the host and poses a lifelong threat of ATL, HAM/TSP, and other inflammatory disorders (10). The mechanism by which HTLV-1 controls viral gene expression and evades immune clearance has not yet been fully elucidated. In this review, we describe persistent HTLV-1 infection and recent findings on the nature of HTLV-1 gene expression. We will discuss the clinical implications of HTLV-1 gene expression in the development of ATL and HAM/TSP.

## Persistence of HTLV-1 Infection

### Global Endemicity and How HTLV-1 Spreads

HTLV-1 is prevalent across the globe. HTLV-1 is particularly endemic in some areas, including southwestern Japan, Central Australia, South America, the Caribbean islands, and sub-Saharan Africa (11). Approximately 5–10 million people are estimated to be infected with HTLV-1 globally (1). HTLV-1 is transmitted *via* infected lymphocytes from HTLV-1 carriers; breastfeeding and sexual contact are common routes of transmission where infectious lymphocytes are transferred to a new host (12–14).

HTLV-1 is mainly found in CD4^+^ T lymphocytes. An infected lymphocyte transmits HTLV-1 through cell-to-cell contact with other lymphocytes. HTLV-1 viral components, including its single-stranded RNA genome, are transferred to target cells through this junction (15). Recently, Hiyoshi et al. reported that the host factor M-Sec plays a critical role in efficient viral transmission (16). M-Sec induces membrane protrusions and establishes intercellular conduits (17). This is likely the molecular basis of what is known as the virological synapse in HTLV-1 infections (15).

HTLV-1 genomic RNA is reverse-transcribed in the target cell, and the resulting double-stranded DNA, 9 kb in size, is inserted into the host genome. The location at which the HTLV-1 provirus is inserted in each infection is not completely random. HTLV-1 favors genomic sites near genes, CpG islands, and chromatin regions with epigenetic marks associated with gene regulation (18). Unlike HIV-1 infection, where the reverse-transcribed HIV-1 genome is guided to actively transcribed genes by the host factor LEDGF (19), the mechanism by which HTLV-1 is preferentially integrated in these characteristic regions is currently unknown. Host factor PP2A has been identified as a binding partner of the HTLV-1 integration complex (20, 21). More studies are needed to elucidate the mechanisms underlying HTLV-1 integration preferences.

### Latent Infection of HTLV-1

It has been postulated that HTLV-1 propagates rapidly in a new host during the early stages of infection. HTLV-1 is believed not to produce cell-free infectious viral particles *in vivo*. HTLV-1 increases the proviral copy number by a combination of *de novo* cell-to-cell infection and mitotic division in each infected cell. Each infected cell carries a single HTLV-1 provirus copy in its genome (22).

The expression of viral genes for HTLV-1 propagation in the new host elicits the host immune response. HTLV-1-infected cells will be lysed by cytotoxic T lymphocytes (CTLs) that are specific for viral antigens (23, 24, 25). Therefore, HTLV-1 propagation is counterbalanced by the host immune response, which in turn determines the set point of proviral load (PVL) in the host. PVL in ACs is approximately 1% [i.e., HTLV-1 is found in 1% of total peripheral blood mononuclear cells (PBMCs)]; PVL varies by 1,000-fold among ACs (26, 27). It is estimated that PVL in each individual is typically maintained by the mitotic division of cells in the chronic phase of infection (28). PVL positively correlates with the risk of developing ATL and HAM/TSP; that is, the risk of disease onset is greater with a higher PVL (26, 29).

HTLV-1 inserts its genome at a unique location on the host chromosome during *de novo* infection. Each infected cell carrying a single copy of the HTLV-1 provirus in the genome gives rise to a group of sister cells, or a clone, by mitotic division, which shares the same proviral integration site. Gillet et al. estimated the abundance of each clone, or clonality, in ACs and patients with ATL and HAM/TSP by quantifying the frequency of each provirus integration site using high-throughput sequencing (30). It is estimated that tens of thousands of unique HTLV-1-infected clones exist in a typical host. These clones persist for many years, from which a malignant clone emerges (31).

### Progression to Diseases

ATL is a malignancy characterized by clonal expansion of HTLV-1-infected lymphocytes, often with a PVL of >90% in acute ATL cases. It takes decades for a malignant clone to emerge from a typical HTLV-1 infection. Recently, two studies retrospectively performed exon sequencing of clinical samples to track gene mutations before ATL onset. These studies found, among other genes, recurrent mutations in CCR4, PLCG1, PRKCB, and NOTCH1 that precede the onset of ATL (32, 33). It is possible that HTLV-1 infection *per se* does not cause ATL. HTLV-1 infection prolongs the lifetime of infected lymphocytes, during which infected lymphocytes acquire a set of gene mutations and undergo malignant transformation.

HAM/TSP is another clinical entity associated with HTLV-1 infections. The PVL is significantly higher in HAM/TSP patients than in ACs (26). Monoclonal expansion is not observed in HAM/TSP; instead, it is envisaged that the number of clones increases, which accounts for the high PVL (30).

HTLV-1 tax and HBZ, as we describe in the next section, are the main viral factors that confer a growth advantage to infected cells. It appears that HTLV-1 performs two contradicting tasks: expressing viral genes to sustain the infected cells and avoiding CTL killing exerted by the host immune response. Therefore, understanding the regulation of HTLV-1 genes *in vivo* is crucial for understanding HTLV-1 infection and its associated diseases.

## Nature of HTLV-1 Gene Expression

### Genomic Structure of HTLV-1

**FIGURE 1:**
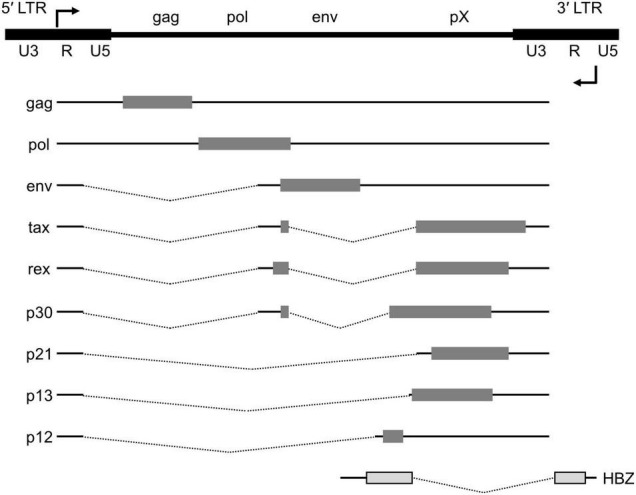
Genome structure of the HTLV-1 provirus. The proviral DNA with the LTRs and viral transcripts encoded in either the plus or minus strand of the viral genome. Alternative splicing yields doubly spliced (tax, rex and p30), singly spliced (env, p21, p13, and p12) mRNAs as well as the unspliced gag and pol transcript. The structural genes (gag, pol and env) and the pX region are flanked by the 5′ and 3′ LTRs. HBZ, encoded on the minus strand, is transcribed from the 3′ LTR.

HTLV-1 viral genes are encoded in both the plus and minus strands of the provirus, which is 9 kb in size and is embedded in the host chromatin (Figure 1). HTLV-1 has two long terminal repeats (LTRs) at the 5′ and 3′ ends of its provirus. HTLV-1 gag, pol, and env, the essential retroviral genes, are encoded on the plus strand. HTLV-1 carries an additional genomic segment, referred to as pX, which is downstream of the env gene. The pX region encodes HTLV-1 tax, rex, and other accessory genes (34). The plus strand is transcribed from the promoter, which resides within the 5′ LTR. Alternative splicing yields mature mRNA for each gene. On the minus strand, HTLV-1 encodes the HTLV-1 bZIP factor, or HBZ, and its transcription is initiated within the 3′ LTR.

Distinct transcription factors operate in plus- and minus-strand transcription. Each LTR consists of three regions: U3, R, and U5. U3 contains binding sites for activating transcription factor (ATF), cAMP response element-binding protein (CREB), and activator protein 1 (AP-1) for plus-strand transcription. In contrast, minus-strand transcription is driven from U5 in the 3′ LTR by transcription factor Sp1 (35). HTLV-1 Tax, once it is produced, forms a complex with the transcription factors on the 5′ LTR and recruits CBP/p300, thereby enhancing viral gene transcription. Recently, another mechanism of plus-strand transcription was reported by Wang et al., where Yin Yang 1 (YY1) binds to the R region of HTLV-1 transcripts and enhances transcription initiation (36). Interestingly, transcriptional enhancement was not observed when the YY1-binding element was placed upstream of the transcription start site. It has been proposed that YY1 binds to HTLV-1 plus-strand transcripts, as opposed to DNA, and enhances transcription initiation.

### HTLV-1 Tax and HBZ

**FIGURE 2:**
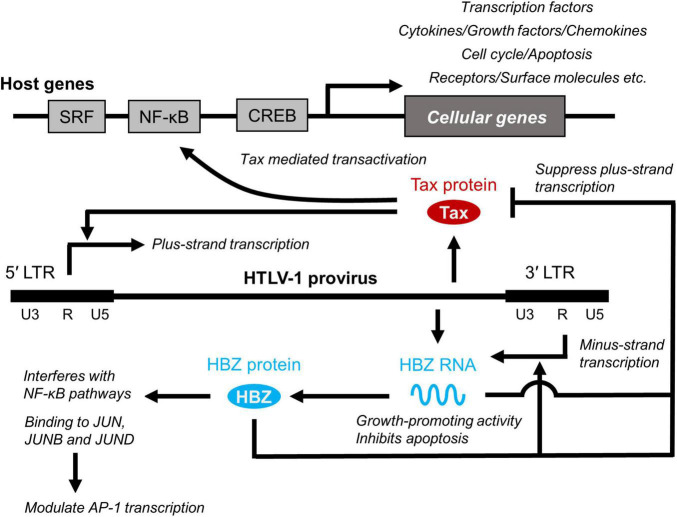
Role of Tax and HBZ. Two viral genes, Tax and HTLV-1 bZIP factor (HBZ) play critical roles in viral transcription and promotion of T-cell proliferation by interacting cellular factors, which is closely related to the long-term survival and persistence of HTLV-1 in infected individuals and development of HTLV-1-associated diseases. SRF, serum response factor.

HTLV-1 tax and HBZ have been extensively studied to understand the pathogenicity of HTLV-1 (37) (Figure 2). Tax binds to several proteins. For example, Tax binds to the nuclear factor κB (NF-κB) components and activates its pathway, resulting in the activation of inflammatory signaling. Conversely, HBZ has a counteracting effect on HTLV-1 tax. HBZ protein interferes with the NF-κB pathway (38). The HBZ protein binds to ATF and AP-1 transcription factors and inhibits their function (39). HBZ RNA also functions in the nucleus. Two recent studies performed RNA precipitation to identify chromatin regions targeted by HBZ RNA. Gazon et al. found that HBZ RNA binds to HTLV-1 LTR and displaces TATA-box binding protein, thereby suppressing the transcription of the plus strand (40). Ma et al. reported that HBZ RNA associates with the CCR4 promoter and enhances CCR4 expression (41). CCR4 is a chemokine receptor highly expressed in ATL (42) and HAM/TSP (43). CCR4 is an important molecule in HTLV-1 infection, not only because its mutation significantly contributes to the development of ATL as described above, but also because it serves as a marker for ATL and HAM/TSP (44, 45), and is targeted by the monoclonal antibody mogamulizumab, a clinically approved drug for the treatment of ATL and HAM/TSP (46, 47).

### Silent Infection of HTLV-1 in PBMCs *in vivo*

Despite the pleiotropic functions of HTLV-1 plus-strand gene tax, tax mRNA is often not detected in clinically isolated PBMCs (48, 49). This observation can be explained by three mechanisms: gene deletion, gene mutation, and DNA methylation near the 5′ LTR, where the plus-strand transcripts are encoded.

HTLV-1 proviruses are often defective in ATL. Tamiya et al. reported two types of defective proviruses: lacking the retroviral gag and pol segments or lacking the 5′ LTR (50). In some ATL cases, the deletion occurs before the HTLV-1 provirus is integrated into the genome (51). A proviral deletion was preferentially found at the 5′ end of the provirus, whereas the 3′ end of the provirus was unaffected. This raises the possibility that plus-strand genes are not essential, whereas the antisense HBZ gene is crucial for pathogenesis (52). More recently, Katsuya et al. reported a gene deletion in the 3′ LTR of the HTLV-1 provirus (53). The significance of losing the 3′ LTR, and hence HBZ expression, on HTLV-1 persistence and pathogenesis is yet to be investigated.

The second mechanism of gene silencing involves point mutation in the tax gene. A point mutation that introduces a premature termination codon in the tax mRNA results in the loss of functional Tax protein, a strong activator of its viral sense promoter (48, 54).

The third mechanism involves epigenetic modification. The cytosine residues in the 5′ LTR and the adjacent downstream region are highly methylated; although the 3′ LTR has a sequence identical to the 5′ LTR, the 3′ LTR is not methylated (55, 56). Currently, it is not known what regulates the contrasting DNA methylation patterns in the 5′ LTR and 3′ LTR. Recently, Satou et al. reported that HTLV-1 binds the host factor CTCF in the pX region upstream of the 3′ LTR (57). CTCF is responsible for transcriptional regulation, DNA insulation, and chromatin folding. Therefore, it was hypothesized that CTCF binding regulates DNA methylation in the pX region and keeps the 3′ LTR open for transcription. Cheng et al. showed that the boundary of DNA methylation moved beyond the CTCF-binding site toward the 3′ LTR without CTCF (58), whereas two CRISPR-mutated primary T cell clones eliminating the CTCF binding on the provirus reported no impact (59). It is possible that the effect of CTCF on regulating DNA methylation depends on the location at which the HTLV-1 provirus is integrated.

### Spontaneous Reactivation of the Plus-Strand Transcription in PBMCs *ex vivo*

Although viral gene expression appears to be silenced during latency in HTLV-1 infection, a strong cellular immune response has been detected for plus-strand products such as Gag and Tax (60). This indicates that plus-strand transcription is not permanently silenced; however, HTLV-1 genes are expressed intermittently, which constantly evokes the host immune response against viral antigens. The apparent silencing of HTLV-1 plus-strand transcription *in vivo* is reversible. HTLV-1-positive PBMCs from HTLV-1-infected individuals initiate viral gene expression once they are isolated from the peripheral blood and cultured (24, 61, 62). Approximately half of HTLV-1-infected PBMCs reactivate plus-strand transcription, although this varies among HTLV-1-infected individuals (20 to 80%) (24, 63). Reactivation occurs rapidly within the first few hours of *in vitro* culture (63, 64). The plus-strand transcription reactivation is intense: about a hundred of transcripts are produced per hour in a single cell with the positive feedback of Tax protein (63). Kulkarni et al. showed that p38 MAP kinase and deubiquitylation of histone H2A in the HTLV-1 provirus are responsible for viral gene activation in *ex vivo* culture (65). The primary stimulation that ultimately leads to the activation and deubiquitylation of these factors is obscure. Any physical or chemical stress that PBMCs experience when drawn from the circulation may trigger spontaneous viral transcription reactivation. It is probable that HTLV-1 reactivates in breast milk in response to non-specific stimulation due to environmental changes.

### Stochastic Transcription of HTLV-1 Genes *in vitro*

Billman et al. recently applied single-molecule RNA fluorescence *in situ* hybridization (FISH) to detect viral transcripts in HTLV-1-infected cells *in vitro* (66). They used HTLV-1-infected cells freshly established and cultured from patient PBMCs (22). Single-molecule FISH detects diffraction-limited spots, each of which are from a single mRNA, thereby allowing for the absolute quantification of viral transcripts expressed in each cell (67). Using this technique, Billman et al. found that plus-strand genes are expressed in a transcription burst. Transcription is rare; however, once the genes are expressed, hundreds of transcripts are produced at a time. In contrast, the minus-strand transcripts contained per cell were much fewer (up to ∼10 molecules). A slight deviation from the Poisson distribution indicates that minus-strand transcription also occurs in a burst, yet it is much smaller. Stochastic HBZ transcription results in approximately 20% of a clonal population with no HBZ transcripts at a given time. The occurrence of the plus-strand transcription burst is associated with the progression to the G2/M cell cycle stage. Although the causation of these two events is not clear, the function of tax and HBZ genes suggests that HTLV-1 gene expression accelerates cell cycle progression.

The termination of a transcriptional burst is a common question in gene regulation. In the study by Billman et al. the occurrence of plus-strand transcription was significantly lower in HBZ-positive cells, in line with other observations that HBZ, in the form of protein (35, 68) or RNA (40), suppresses plus-strand expression.

### Conundrums in HTLV-1 Gene Expression *in vivo* and *in vitro*

It has been postulated that HTLV-1 genes are intermittently expressed *in vivo*. Does the rare expression of HTLV-1 plus-strand genes observed *in vitro* account for the viral expression *in vivo*? Therefore, if, and when PBMCs intermittently transcribe HTLV-1 plus-strand genes *in vivo*, are the frequency and intensity of expression similar to those observed *in vitro*? In a previous study where single-molecule RNA FISH was performed on hundreds to thousands of HTLV-1-infected PBMCs for each HTLV-1-positive subject, no intense plus-strand transcription burst was reported unless cultured *in vitro* (63). The frequency and intensity of plus-strand gene bursts *in vivo* should be much smaller than those observed *in vitro*.

There is an apparent discrepancy between PBMCs *in vivo*, fresh *in vitro* culture, and HTLV-1-infected cells maintained *in vitro*. Is it possible to translate the findings of HTLV-1 gene expression *in vitro* into the unseen nature of HTLV-1 gene expression *in vivo*?

First, there is a correlation between spontaneous plus-strand reactivation in HTLV-1-positive PBMCs *in vitro* and the expression of HTLV-1 genes *in vivo*. Patient-derived PBMCs contain many distinct clones of HTLV-1-infected lymphocytes. The provirus integration site is a strong determinant of spontaneous plus-strand transcription *in vitro*, and the degree of spontaneous expression *in vitro* is inversely correlated with the clonal abundance (18). This indicates that a clone that reactivates plus-strand transcription *in vitro* also transcribes HTLV-1 genes at high frequency *in vivo*, as the relatively small abundance of that clone is a result of CTL killing that recognizes viral expression (24).

It appears that cells *in vitro*, where a strong plus-strand transcription burst is observed, are in another equilibrium state that is different from what might otherwise be *in vivo* in which HTLV-1 is silenced. HTLV-1-positive PBMCs show transient, spontaneous reactivation of plus-strand transcription along the way throughout the circulation. It is possible that HTLV-1-positive lymphocytes express plus-strand genes when certain conditions are met *in vivo*, if not in peripheral blood, such as in lymph nodes or bone marrow (69), especially with the aid of local stimulatory signals from other cells in those compartments. This possibility is supported by the *in vitro* study by Kulkarni et al. that lower glucose availability and hypoxic conditions both enhance tax transcription (70). We are currently developing a microscopic technique to capture the transcription burst in each HTLV-1-positive clone in a given native tissue environment.

Finally, is the spontaneous expression of viral genes truly stochastic? If it is truly stochastic, then the expression is governed by the probabilistic binding of biochemical molecules under random thermodynamic fluctuations. Or if it is not otherwise, there should be unseen factors that determine the HTLV-1 gene expression. It is tempting to assay the transcription burst on an HTLV-1-infected cell line carrying multiple copies of the HTLV-1 provirus: if the multiple HTLV-1 copies burst at the same instance within a single cell, then this predicts that there are unseen factors that coordinate the HTLV-1 transcription initiation. The outcome of the *in vitro* study will be translated into an understanding of how HTLV-1 gene expression is regulated *in vivo*.

## Clinical Implications of the HTLV-1 Gene Expression

### Overview

**FIGURE 3:**
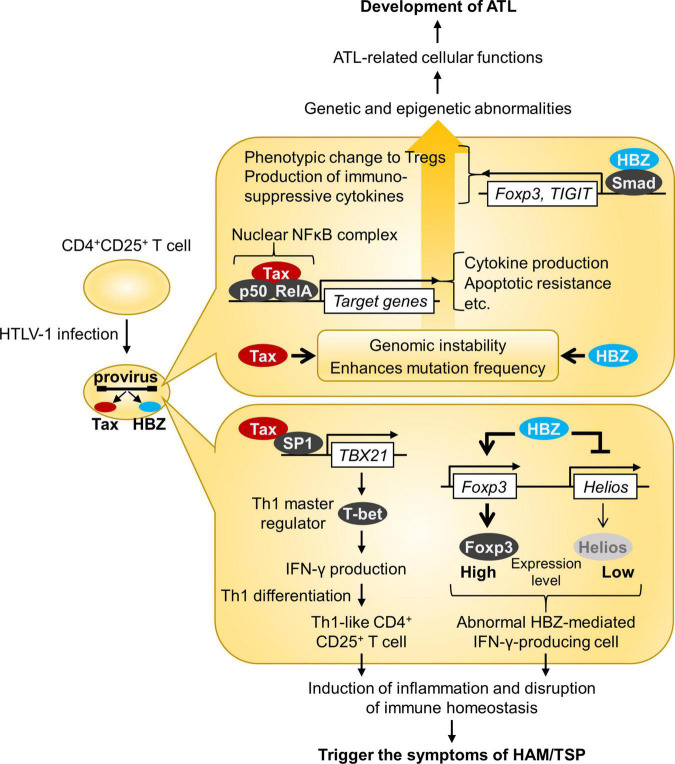
Roles of HTLV-1 Tax and HBZ in the pathogenesis of ATL and HAM/TSP. This figure illustrates the model for ATL and HAM/TSP development. Both Tax and HBZ play crucial roles in oncogenic and inflammatory processes through multiple mechanisms.

As HTLV-1 transmission requires cell contact, HTLV-1 propagates within the host by both clonal expansion of infected cells and *de novo* viral infection. In HTLV-1-infected individuals, cell-free virus particles are usually undetectable, and the plasma does not transmit the infection. Furthermore, PVL in PBMCs, which reflects the number of virus-infected cells, correlates with the risk of developing ATL and HAM/TSP (26, 29). It is therefore believed that HTLV-1 is almost entirely cell-associated *in vivo*, and clonal proliferation of infected cells predisposes individuals to ATL and HAM/TSP. Among HTLV-1 genes, tax and HBZ play a particularly important role in regulating the expression of viral and host genes as well as the activation and proliferation of host cells (71) (Figure 2). Tax induces the expression of serum response factor (SRF) and various cellular genes *via* transcriptional pathways, such as the NF-κB, CREB, and AP-1 pathways (72, 73). In contrast, the HBZ protein suppresses the transcription of the tax gene and the cellular pathways that Tax activates. HBZ RNA suppresses apoptosis by inducing survivin expression (74) and, therefore, promotes the proliferation of T cells (52). Thus, understanding how HTLV-1 regulates the expression of viral and cellular genes *in vivo* is key to elucidating the mechanisms of long-term survival and the persistence of HTLV-1 in infected individuals, which is closely related to the development of HTLV-1-associated diseases. The roles of Tax and HBZ in the pathogenesis of ATL and HAM/TSP are summarized in Figure 3.

### Clinical Implications of the HTLV-1 Gene Expression in ATL

Approximately 60% of ATL patients do not express tax mRNA in freshly isolated PBMCs (48). Tax is often repressed once ATL develops (75), whereas HBZ mRNA is expressed in all ATL cases (49), because HTLV-1 provirus is substantially silenced by proviral defects and/or epigenetic mechanisms (see section “Silent Infection of HTLV-1 in PBMCs *in vivo”*). These findings suggest that Tax is essential to initiate transformation, while HBZ has roles in promoting viral replication and cellular proliferation to maintain the transformed ATL cells when Tax expression is extinguished. If this is the case, it may be the most efficient method to escape HTLV-1-specific CTLs.

Previous reports have described the downregulation of microRNAs in ATL cells (76, 77). This may cause disordered gene expression at the transcriptional and post-transcriptional levels, thereby contributing to the development of ATL. The relationship between downregulation of microRNA and gene expression of tax and/or HBZ has not been reported; thus, it should be investigated in future studies.

### Clinical Implications of the HTLV-1 Gene Expression in HAM/TSP

In patients with HAM/TSP, the quantity of PVL in PBMCs is significantly higher than that in ACs and is well correlated with the concentration of neopterin in the cerebrospinal fluid (CSF) (26), a marker associated with cell-mediated immunity (78), and with disease progression (79). In individual HAM/TSP patients, PVL in CSF cells was higher than that in PBMCs, and the ratio of PVLs in CSF cells/PBMCs was significantly associated with clinically progressive disease and recent onset of HAM/TSP (80). Thus, HTLV-1 PVL is an important biomarker for HAM/TSP. Meanwhile, the total amount of HTLV-1 tax mRNA in PBMCs and mRNA expression level in HTLV-1-infected cells (mRNA/DNA ratio) were significantly higher in HAM/TSP patients than in ACs and correlated with PVL, Tax-specific CTL frequency, and disease severity of the patients (81). In addition, HBZ mRNA load was positively correlated with PVL, disease severity, and neopterin concentration in the CSF of HAM/TSP patients (82).

HTLV-1-specific CTLs are abundant in PBMCs of infected individuals, and their frequency is proportional to the PVL, indicating that HTLV-1 is not latent *in vivo* but is expressed persistently or at frequent intervals in infected individuals (3). Interestingly, in HTLV-1 infection, although the dominant antigen recognized by HTLV-1-specific CTLs is the Tax protein (83, 84), PVL and the risk of HAM/TSP are determined by the CTL response to poorly immunogenic HBZ proteins (85, 86). This is consistent with the idea that a persistent HTLV-1 infection establishes an equilibrium between viral replication and the host immune response, and that the response of HTLV-1 specific CTLs determines the equilibrium PVL and the risk of HAM/TSP. Meanwhile, it remains possible that the chronically activated anti-HTLV-1 CTLs found in patients with HAM/TSP contribute to systemic inflammation. Many host genes dysregulated by Tax and HBZ may activate and proliferate host cells and induce systemic inflammation *in vivo*.

### Inflammation and Tumorigenesis

In recent decades, it has become evident that chronic inflammation and tumor development are closely related (87). Infection, chemical substances, and injury can initiate tumorigenesis at the associated inflammation site. Alternatively, tumor induction triggers inflammation through the secretion of chemokines and inflammatory signaling molecules, creating a local microenvironment that supports further tumor development.

There is strong evidence suggesting that inflammation, induced by HBZ, promotes the development of ATL. HBZ-transgenic mice develop lymphoma, in which it was shown that HBZ induces Foxp3 expression and the proliferation of the regulatory T-cell phenotype (88). However, the Foxp3 expression in these cells is not stable; such cells secrete IFN-γ (Interferon-gamma) and promote systemic chronic inflammation (89). The incidence of lymphoma was significantly low in HBZ-transgenic mice lacking IFN-γ, suggesting that IFN-γ, alongside HBZ, promotes tumorigenesis in HTLV-1 infection (90). More recently, Higuchi et al. reported an unexpected observation; the deletion of IL-6, also a pro-inflammatory cytokine, increased the incidence of lymphoma development in their transgenic mice, and IL-10 was upregulated in HBZ-transgenic mice lacking IL-6 (91). IL-10 is an immunosuppressive cytokine and it is known to promote the proliferation of HTLV-1-infected T cells *in vitro* (92). Higuchi et al. have shown that IL-10 signaling is redirected to T-cell proliferation by HBZ modulating the STAT pathway.

## Concluding Remarks

HTLV-1 induces T-cell leukemia/lymphoma and systemic inflammation *in vivo*. Increasing evidence suggests that both HBZ and Tax play distinct but important roles during very long latency periods in disease induction. Characterization of the viral gene expression profile throughout the infection process is essential to provide key functional information to shed light on HTLV-1 pathogenesis. As ATL is still dismal and HAM/TSP remains an intractable disease, the establishment of a precise understanding of disease developmental pathways is an urgent requirement. Further studies using newly developed methods with large amounts of data, such as computational biology and bioinformatics, are warranted to provide *in vivo* evidence for these points.

## Author Contributions

MM, TN, and MS analyzed past literature and wrote the manuscript. All authors contributed to the article and approved the submitted version.

## Conflict of Interest

The authors declare that the research was conducted in the absence of any commercial or financial relationships that could be construed as a potential conflict of interest.

## Publisher’s Note

All claims expressed in this article are solely those of the authors and do not necessarily represent those of their affiliated organizations, or those of the publisher, the editors and the reviewers. Any product that may be evaluated in this article, or claim that may be made by its manufacturer, is not guaranteed or endorsed by the publisher.

## References

[1] Gessain A Cassar O. Epidemiological aspects and world distribution of HTLV-1 infection. *Front Microbiol.* (2012) 3:388. 10.3389/fmicb.2012.00388 23162541 PMC3498738

[2] Iwanaga M Watanabe T Yamaguchi K. Adult T-cell leukemia: a review of epidemiological evidence. *Front Microbiol.* (2012) 3:322. 10.3389/fmicb.2012.00322 22973265 PMC3437524

[3] Saito M. Neuroimmunological aspects of human T cell leukemia virus type 1-associated myelopathy/tropical spastic paraparesis. *J Neurovirol.* (2014) 20:164–74. 10.1007/s13365-013-0192-8 23943469

[4] Sato T Yagishita N Tamaki K Inoue E Hasegawa D Nagasaka M Proposal of classification criteria for HTLV-1-associated myelopathy/tropical spastic paraparesis disease activity. *Front Microbiol.* (2018) 9:1651. 10.3389/fmicb.2018.01651 30090093 PMC6068401

[5] Poiesz BJ Ruscetti FW Gazdar AF Bunn PA Minna JD Gallo RC. Detection and isolation of type C retrovirus particles from fresh and cultured lymphocytes of a patient with cutaneous T-cell lymphoma. *Proc Natl Acad Sci USA.* (1980) 77:7415–9. 10.1073/pnas.77.12.7415 6261256 PMC350514

[6] Gessain A Barin F Vernant JC Gout O Maurs L Calender A Antibodies to human T-lymphotropic virus type-I in patients with tropical spastic paraparesis. *Lancet.* (1985) 2:407–10. 10.1016/s0140-6736(85)92734-5 2863442

[7] Osame M Usuku K Izumo S Ijichi N Amitani H Igata A HTLV-I associated myelopathy, a new clinical entity. *Lancet.* (1986) 1:1031–2. 10.1016/s0140-6736(86)91298-5 2871307

[8] Katsuya H Ishitsuka K Utsunomiya A Hanada S Eto T Moriuchi Y Treatment and survival among 1594 patients with ATL. *Blood.* (2015) 126:2570–7. 10.1182/blood-2015-03-632489 26361794

[9] Araujo A Bangham CRM Casseb J Gotuzzo E Jacobson S Martin F Management of HAM/TSP: systematic review and consensus-based recommendations 2019. *Neurol Clin Pract.* (2021) 11:49–56. 10.1212/CPJ.0000000000000832 33968472 PMC8101298

[10] Bangham CRM Miura M Kulkarni A Matsuoka M. Regulation of latency in the human T cell leukemia virus, HTLV-1. *Annu Rev Virol.* (2019) 6:365–85. 10.1146/annurev-virology-092818-015501 31283437

[11] Verdonck K Gonzalez E Van Dooren S Vandamme AM Vanham G Gotuzzo E. Human T-lymphotropic virus 1: recent knowledge about an ancient infection. *Lancet Infect Dis.* (2007) 7:266–81. 10.1016/S1473-3099(07)70081-6 17376384

[12] Paiva A Casseb J. Sexual transmission of human T-cell lymphotropic virus type 1. *Rev Soc Bras Med Trop.* (2014) 47:265–74. 10.1590/0037-8682-0232-2013 25075475

[13] Satake M Iwanaga M Sagara Y Watanabe T Okuma K Hamaguchi I. Incidence of human T-lymphotropic virus 1 infection in adolescent and adult blood donors in Japan: a nationwide retrospective cohort analysis. *Lancet Infect Dis.* (2016) 16:1246–54. 10.1016/S1473-3099(16)30252-3 27567105

[14] Rosadas C Taylor GP. Mother-to-child HTLV-1 transmission: unmet research needs. *Front Microbiol.* (2019) 10:999. 10.3389/fmicb.2019.00999 31134031 PMC6517543

[15] Igakura T Stinchcombe JC Goon PK Taylor GP Weber JN Griffiths GM Spread of HTLV-I between lymphocytes by virus-induced polarization of the cytoskeleton. *Science.* (2003) 299:1713–6. 10.1126/science.1080115 12589003

[16] Hiyoshi M Takahashi N Eltalkhawy YM Noyori O Lotfi S Panaampon J M-Sec induced by HTLV-1 mediates an efficient viral transmission. *PLoS Pathog.* (2021) 17:e1010126. 10.1371/journal.ppat.1010126 34843591 PMC8659635

[17] Hase K Kimura S Takatsu H Ohmae M Kawano S Kitamura H M-Sec promotes membrane nanotube formation by interacting with Ral and the exocyst complex. *Nat Cell Biol.* (2009) 11:1427–32. 10.1038/ncb1990 19935652

[18] Melamed A Laydon DJ Gillet NA Tanaka Y Taylor GP Bangham CR. Genome-wide determinants of proviral targeting, clonal abundance and expression in natural HTLV-1 infection. *PLoS Pathog.* (2013) 9:e1003271. 10.1371/journal.ppat.1003271 23555266 PMC3605240

[19] Engelman A Cherepanov P. The lentiviral integrase binding protein LEDGF/p75 and HIV-1 replication. *PLoS Pathog.* (2008) 4:e1000046. 10.1371/journal.ppat.1000046 18369482 PMC2275779

[20] Maertens GN. B’-protein phosphatase 2A is a functional binding partner of delta-retroviral integrase. *Nucleic Acids Res.* (2016) 44:364–76. 10.1093/nar/gkv1347 26657642 PMC4705670

[21] Barski MS Minnell JJ Hodakova Z Pye VE Nans A Cherepanov P Cryo-EM structure of the deltaretroviral intasome in complex with the PP2A regulatory subunit B56gamma. *Nat Commun.* (2020) 11:5043. 10.1038/s41467-020-18874-y 33028863 PMC7542444

[22] Cook LB Rowan AG Melamed A Taylor GP Bangham CR. HTLV-1-infected T cells contain a single integrated provirus in natural infection. *Blood.* (2012) 120:3488–90. 10.1182/blood-2012-07-445593 22955925 PMC3482858

[23] Jacobson S Shida H Mcfarlin DE Fauci AS Koenig S. Circulating CD8+ cytotoxic T lymphocytes specific for HTLV-I pX in patients with HTLV-I associated neurological disease. *Nature.* (1990) 348:245–8. 10.1038/348245a0 2146511

[24] Hanon E Hall S Taylor GP Saito M Davis R Tanaka Y Abundant tax protein expression in CD4+ T cells infected with human T-cell lymphotropic virus type I (HTLV-I) is prevented by cytotoxic T lymphocytes. *Blood.* (2000) 95:1386–92. 10.1182/blood.v95.4.1386.004k22_1386_1392 10666215

[25] Hanon E Stinchcombe JC Saito M Asquith BE Taylor GP Tanaka Y Fratricide among CD8(+) T lymphocytes naturally infected with human T cell lymphotropic virus type I. *Immunity.* (2000) 13:657–64. 10.1016/s1074-7613(00)00065-0 11114378

[26] Nagai M Usuku K Matsumoto W Kodama D Takenouchi N Moritoyo T Analysis of HTLV-I proviral load in 202 HAM/TSP patients and 243 asymptomatic HTLV-I carriers: high proviral load strongly predisposes to HAM/TSP. *J Neurovirol.* (1998) 4:586–93. 10.3109/13550289809114225 10065900

[27] Demontis MA Sadiq MT Golz S Taylor GP. HTLV-1 viral RNA is detected rarely in plasma of HTLV-1 infected subjects. *J Med Virol.* (2015) 87:2130–4. 10.1002/jmv.24264 25982784

[28] Laydon DJ Sunkara V Boelen L Bangham CRM Asquith B. The relative contributions of infectious and mitotic spread to HTLV-1 persistence. *PLoS Comput Biol.* (2020) 16:e1007470. 10.1371/journal.pcbi.1007470 32941445 PMC7524007

[29] Iwanaga M Watanabe T Utsunomiya A Okayama A Uchimaru K Koh KR Human T-cell leukemia virus type I (HTLV-1) proviral load and disease progression in asymptomatic HTLV-1 carriers: a nationwide prospective study in Japan. *Blood.* (2010) 116:1211–9. 10.1182/blood-2009-12-257410 20448111

[30] Gillet NA Malani N Melamed A Gormley N Carter R Bentley D The host genomic environment of the provirus determines the abundance of HTLV-1-infected T-cell clones. *Blood.* (2011) 117:3113–22. 10.1182/blood-2010-10-312926 21228324 PMC3062313

[31] Cook LB Melamed A Niederer H Valganon M Laydon D Foroni L The role of HTLV-1 clonality, proviral structure, and genomic integration site in adult T-cell leukemia/lymphoma. *Blood.* (2014) 123:3925–31. 10.1182/blood-2014-02-553602 24735963 PMC4064332

[32] Rowan AG Dillon R Witkover A Melamed A Demontis MA Gillet NA Evolution of retrovirus-infected premalignant T-cell clones prior to adult T-cell leukemia/lymphoma diagnosis. *Blood.* (2020) 135:2023–32. 10.1182/blood.2019002665 32160278 PMC7381760

[33] Yamagishi M Kubokawa M Kuze Y Suzuki A Yokomizo A Kobayashi S Chronological genome and single-cell transcriptome integration characterizes the evolutionary process of adult T cell leukemia-lymphoma. *Nat Commun.* (2021) 12:4821. 10.1038/s41467-021-25101-9 34376672 PMC8355240

[34] Matsuoka M Jeang KT. Human T-cell leukaemia virus type 1 (HTLV-1) infectivity and cellular transformation. *Nat Rev Cancer.* (2007) 7:270–80. 10.1038/nrc2111 17384582

[35] Yoshida M Satou Y Yasunaga J Fujisawa J Matsuoka M. Transcriptional control of spliced and unspliced human T-cell leukemia virus type 1 bZIP factor (HBZ) gene. *J Virol.* (2008) 82:9359–68. 10.1128/JVI.00242-08 18653454 PMC2546946

[36] Wang GZ Goff SP. Yin Yang 1 is a potent activator of human T lymphotropic virus type 1 LTR-driven gene expression via RNA binding. *Proc Natl Acad Sci USA.* (2020) 117:18701–10. 10.1073/pnas.2005726117 32690679 PMC7414092

[37] Matsuoka M Yasunaga J. Human T-cell leukemia virus type 1: replication, proliferation and propagation by Tax and HTLV-1 bZIP factor. *Curr Opin Virol.* (2013) 3:684–91. 10.1016/j.coviro.2013.08.010 24060211

[38] Zhao T Yasunaga J Satou Y Nakao M Takahashi M Fujii M Human T-cell leukemia virus type 1 bZIP factor selectively suppresses the classical pathway of NF-kappaB. *Blood.* (2009) 113:2755–64. 10.1182/blood-2008-06-161729 19064727

[39] Basbous J Arpin C Gaudray G Piechaczyk M Devaux C Mesnard JM. The HBZ factor of human T-cell leukemia virus type I dimerizes with transcription factors JunB and c-Jun and modulates their transcriptional activity. *J Biol Chem.* (2003) 278:43620–7. 10.1074/jbc.M307275200 12937177

[40] Gazon H Chauhan PS Porquet F Hoffmann GB Accolla R Willems L. Epigenetic silencing of HTLV-1 expression by the HBZ RNA through interference with the basal transcription machinery. *Blood Adv.* (2020) 4:5574–9. 10.1182/bloodadvances.2020001675 33170933 PMC7656924

[41] Ma G Yasunaga JI Shimura K Takemoto K Watanabe M Amano M Human retroviral antisense mRNAs are retained in the nuclei of infected cells for viral persistence. *Proc Natl Acad Sci USA.* (2021) 118:e2014783118. 10.1073/pnas.2014783118 33875584 PMC8092383

[42] Yoshie O Fujisawa R Nakayama T Harasawa H Tago H Izawa D Frequent expression of CCR4 in adult T-cell leukemia and human T-cell leukemia virus type 1-transformed T cells. *Blood.* (2002) 99:1505–11. 10.1182/blood.v99.5.1505 11861261

[43] Yamano Y Araya N Sato T Utsunomiya A Azakami K Hasegawa D Abnormally high levels of virus-infected IFN-gamma+ CCR4+ CD4+ CD25+ T cells in a retrovirus-associated neuroinflammatory disorder. *PLoS One.* (2009) 4:e6517. 10.1371/journal.pone.0006517 19654865 PMC2715877

[44] Araya N Sato T Ando H Tomaru U Yoshida M Coler-Reilly A HTLV-1 induces a Th1-like state in CD4+CCR4+ T cells. *J Clin Invest.* (2014) 124:3431–42. 10.1172/jci75250 24960164 PMC4109535

[45] Sugata K Yasunaga J Kinosada H Mitobe Y Furuta R Mahgoub M HTLV-1 viral factor HBZ induces CCR4 to promote T-cell migration and proliferation. *Cancer Res.* (2016) 76:5068–79. 10.1158/0008-5472.CAN-16-0361 27402079

[46] Yamauchi J Coler-Reilly A Sato T Araya N Yagishita N Ando H Mogamulizumab, an anti-CCR4 antibody, targets human T-lymphotropic virus type 1-infected CD8+ and CD4+ T cells to treat associated myelopathy. *J Infect Dis.* (2015) 211:238–48. 10.1093/infdis/jiu438 25104771

[47] Sato T Coler-Reilly ALG Yagishita N Araya N Inoue E Furuta R Mogamulizumab (Anti-CCR4) in HTLV-1-associated myelopathy. *N Engl J Med.* (2018) 378:529–38. 10.1056/nejmoa1704827 29414279

[48] Takeda S Maeda M Morikawa S Taniguchi Y Yasunaga J Nosaka K Genetic and epigenetic inactivation of tax gene in adult T-cell leukemia cells. *Int J Cancer.* (2004) 109:559–67. 10.1002/ijc.20007 14991578

[49] Satou Y Yasunaga J Yoshida M Matsuoka M. HTLV-I basic leucine zipper factor gene mRNA supports proliferation of adult T cell leukemia cells. *Proc Natl Acad Sci USA.* (2006) 103:720–5. 10.1073/pnas.0507631103 16407133 PMC1334651

[50] Tamiya S Matsuoka M Etoh K Watanabe T Kamihira S Yamaguchi K Two types of defective human T-lymphotropic virus type I provirus in adult T-cell leukemia. *Blood.* (1996) 88:3065–73. 10.1182/blood.v88.8.3065.bloodjournal8883065 8874205

[51] Miyazaki M Yasunaga J Taniguchi Y Tamiya S Nakahata T Matsuoka M. Preferential selection of human T-cell leukemia virus type 1 provirus lacking the 5′ long terminal repeat during oncogenesis. *J Virol.* (2007) 81:5714–23. 10.1128/JVI.02511-06 17344291 PMC1900290

[52] Matsuoka M Mesnard JM. HTLV-1 bZIP factor: the key viral gene for pathogenesis. *Retrovirology.* (2020) 17:2. 10.1186/s12977-020-0511-0 31915026 PMC6950816

[53] Katsuya H Islam S Tan BJY Ito J Miyazato P Matsuo M The nature of the HTLV-1 provirus in naturally infected individuals analyzed by the viral DNA-capture-seq approach. *Cell Rep.* (2019) 29:724–35.e724. 10.1016/j.celrep.2019.09.016 31618639

[54] Furukawa Y Kubota R Tara M Izumo S Osame M. Existence of escape mutant in HTLV-I tax during the development of adult T-cell leukemia. *Blood.* (2001) 97:987–93. 10.1182/blood.v97.4.987 11159527

[55] Koiwa T Hamano-Usami A Ishida T Okayama A Yamaguchi K Kamihira S 5′-long terminal repeat-selective CpG methylation of latent human T-cell leukemia virus type 1 provirus in vitro and in vivo. *J Virol.* (2002) 76:9389–97. 10.1128/jvi.76.18.9389-9397.2002 12186921 PMC136445

[56] Taniguchi Y Nosaka K Yasunaga J Maeda M Mueller N Okayama A Silencing of human T-cell leukemia virus type I gene transcription by epigenetic mechanisms. *Retrovirology.* (2005) 2:64. 10.1186/1742-4690-2-64 16242045 PMC1289293

[57] Satou Y Miyazato P Ishihara K Yaguchi H Melamed A Miura M The retrovirus HTLV-1 inserts an ectopic CTCF-binding site into the human genome. *Proc Natl Acad Sci USA.* (2016) 113:3054–9. 10.1073/pnas.1423199113 26929370 PMC4801255

[58] Cheng X Joseph A Castro V Chen-Liaw A Skidmore Z Ueno T Epigenomic regulation of human T-cell leukemia virus by chromatin-insulator CTCF. *PLoS Pathog.* (2021) 17:e1009577. 10.1371/journal.ppat.1009577 34019588 PMC8174705

[59] Miura M Miyazato P Satou Y Tanaka Y Bangham CRM. Epigenetic changes around the pX region and spontaneous HTLV-1 transcription are CTCF-independent. *Wellcome Open Res.* (2018) 3:105. 10.12688/wellcomeopenres.14741.2 30607369 PMC6305241

[60] Bangham CR Osame M. Cellular immune response to HTLV-1. *Oncogene.* (2005) 24:6035–46. 10.1038/sj.onc.1208970 16155610

[61] Tochikura T Iwahashi M Matsumoto T Koyanagi Y Hinuma Y Yamamoto N. Effect of human serum anti-HTLV antibodies on viral antigen induction in vitro cultured peripheral lymphocytes from adult T-cell leukemia patients and healthy virus carriers. *Int J Cancer.* (1985) 36:1–7. 10.1002/ijc.2910360102 2862107

[62] Minato S Itoyama Y Goto I Yamamoto N. Expression of HTLV-I antigen in cultured peripheral blood mononuclear cells from patients with HTLV-I associated myelopathy. *J Neurol Sci.* (1988) 87:233–44. 10.1016/0022-510x(88)90248-1 3210035

[63] Miura M Dey S Ramanayake S Singh A Rueda DS Bangham CRM. Kinetics of HTLV-1 reactivation from latency quantified by single-molecule RNA FISH and stochastic modelling. *PLoS Pathog.* (2019) 15:e1008164. 10.1371/journal.ppat.1008164 31738810 PMC6886867

[64] Rende F Cavallari I Corradin A Silic-Benussi M Toulza F Toffolo GM Kinetics and intracellular compartmentalization of HTLV-1 gene expression: nuclear retention of HBZ mRNAs. *Blood.* (2011) 117:4855–9. 10.1182/blood-2010-11-316463 21398577 PMC5292588

[65] Kulkarni A Taylor GP Klose RJ Schofield CJ Bangham CR. Histone H2A monoubiquitylation and p38-MAPKs regulate immediate-early gene-like reactivation of latent retrovirus HTLV-1. *JCI Insight.* (2018) 3:e123196. 10.1172/jci.insight.123196 30333309 PMC6237452

[66] Billman MR Rueda D Bangham CRM. Single-cell heterogeneity and cell-cycle-related viral gene bursts in the human leukaemia virus HTLV-1. *Wellcome Open Res.* (2017) 2:87. 10.12688/wellcomeopenres.12469.2 29062917 PMC5645716

[67] Femino AM Fay FS Fogarty K Singer RH. Visualization of single RNA transcripts in situ. *Science.* (1998) 280:585–90. 10.1126/science.280.5363.585 9554849

[68] Clerc I Polakowski N Andre-Arpin C Cook P Barbeau B Mesnard JM An interaction between the human T cell leukemia virus type 1 basic leucine zipper factor (HBZ) and the KIX domain of p300/CBP contributes to the down-regulation of tax-dependent viral transcription by HBZ. *J Biol Chem.* (2008) 283:23903–13. 10.1074/jbc.M803116200 18599479 PMC3259792

[69] Furuta R Yasunaga JI Miura M Sugata K Saito A Akari H Human T-cell leukemia virus type 1 infects multiple lineage hematopoietic cells in vivo. *PLoS Pathog.* (2017) 13:e1006722. 10.1371/journal.ppat.1006722 29186194 PMC5724899

[70] Kulkarni A Mateus M Thinnes CC Mccullagh JS Schofield CJ Taylor GP Glucose metabolism and oxygen availability govern reactivation of the latent human retrovirus HTLV-1. *Cell Chem Biol.* (2017) 24:1377–87.e1373. 10.1016/j.chembiol.2017.08.016 28965728 PMC5696563

[71] Matsuoka M Jeang KT. Human T-cell leukemia virus type 1 (HTLV-1) and leukemic transformation: viral infectivity, Tax, HBZ and therapy. *Oncogene.* (2011) 30:1379–89. 10.1038/onc.2010.537 21119600 PMC3413891

[72] Yoshida M. Multiple viral strategies of HTLV-1 for dysregulation of cell growth control. *Annu Rev Immunol.* (2001) 19:475–96. 10.1146/annurev.immunol.19.1.475 11244044

[73] Franchini G Fukumoto R Fullen JR. T-cell control by human T-cell leukemia/lymphoma virus type 1. *Int J Hematol.* (2003) 78:280–96. 10.1007/bf02983552 14686485

[74] Mitobe Y Yasunaga J Furuta R Matsuoka M. HTLV-1 bZIP factor RNA and protein impart distinct functions on t-cell proliferation and survival. *Cancer Res.* (2015) 75:4143–52. 10.1158/0008-5472.CAN-15-0942 26383166

[75] Kinoshita T Shimoyama M Tobinai K Ito M Ito S Ikeda S Detection of mRNA for the tax1/rex1 gene of human T-cell leukemia virus type I in fresh peripheral blood mononuclear cells of adult T-cell leukemia patients and viral carriers by using the polymerase chain reaction. *Proc Natl Acad Sci USA.* (1989) 86:5620–4. 10.1073/pnas.86.14.5620 2787512 PMC297674

[76] Sampey GC Van Duyne R Currer R Das R Narayanan A Kashanchi F. Complex role of microRNAs in HTLV-1 infections. *Front Genet.* (2012) 3:295. 10.3389/fgene.2012.00295 23251140 PMC3523292

[77] Yamagishi M Nakano K Miyake A Yamochi T Kagami Y Tsutsumi A Polycomb-mediated loss of miR-31 activates NIK-dependent NF-kappaB pathway in adult T cell leukemia and other cancers. *Cancer Cell.* (2012) 21:121–35. 10.1016/j.ccr.2011.12.015 22264793

[78] Huber C Batchelor JR Fuchs D Hausen A Lang A Niederwieser D Immune response-associated production of neopterin. Release from macrophages primarily under control of interferon-gamma. *J Exp Med.* (1984) 160:310–6. 10.1084/jem.160.1.310 6429267 PMC2187425

[79] Sato T Coler-Reilly A Utsunomiya A Araya N Yagishita N Ando H CSF CXCL10, CXCL9, and neopterin as candidate prognostic biomarkers for HTLV-1-associated myelopathy/tropical spastic paraparesis. *PLoS Negl Trop Dis.* (2013) 7:e2479. 10.1371/journal.pntd.0002479 24130912 PMC3794911

[80] Takenouchi N Yamano Y Usuku K Osame M Izumo S. Usefulness of proviral load measurement for monitoring of disease activity in individual patients with human T-lymphotropic virus type I-associated myelopathy/tropical spastic paraparesis. *J Neurovirol.* (2003) 9:29–35. 10.1080/13550280390173418 12587066

[81] Yamano Y Nagai M Brennan M Mora CA Soldan SS Tomaru U Correlation of human T-cell lymphotropic virus type 1 (HTLV-1) mRNA with proviral DNA load, virus-specific CD8(+) T cells, and disease severity in HTLV-1-associated myelopathy (HAM/TSP). *Blood.* (2002) 99:88–94. 10.1182/blood.v99.1.88 11756157

[82] Saito M Matsuzaki T Satou Y Yasunaga J Saito K Arimura K In vivo expression of the HBZ gene of HTLV-1 correlates with proviral load, inflammatory markers and disease severity in HTLV-1 associated myelopathy/tropical spastic paraparesis (HAM/TSP). *Retrovirology.* (2009) 6:19. 10.1186/1742-4690-6-19 19228429 PMC2653460

[83] Kannagi M Harada S Maruyama I Inoko H Igarashi H Kuwashima G Predominant recognition of human T cell leukemia virus type I (HTLV-I) pX gene products by human CD8+ cytotoxic T cells directed against HTLV-I-infected cells. *Int Immunol.* (1991) 3:761–7. 10.1093/intimm/3.8.761 1911545

[84] Goon PK Biancardi A Fast N Igakura T Hanon E Mosley AJ Human T cell lymphotropic virus (HTLV) type-1-specific CD8+ T cells: frequency and immunodominance hierarchy. *J Infect Dis.* (2004) 189:2294–8. 10.1086/420832 15181578

[85] Macnamara A Rowan A Hilburn S Kadolsky U Fujiwara H Suemori K HLA class I binding of HBZ determines outcome in HTLV-1 infection. *PLoS Pathog.* (2010) 6:e1001117. 10.1371/journal.ppat.1001117 20886101 PMC2944806

[86] Hilburn S Rowan A Demontis MA Macnamara A Asquith B Bangham CR In vivo expression of human T-lymphotropic virus type 1 basic leucine-zipper protein generates specific CD8+ and CD4+ T-lymphocyte responses that correlate with clinical outcome. *J Infect Dis.* (2011) 203:529–36. 10.1093/infdis/jiq078 21208912 PMC3071236

[87] Greten FR Grivennikov SI. Inflammation and cancer: triggers, mechanisms, and consequences. *Immunity.* (2019) 51:27–41. 10.1016/j.immuni.2019.06.025 31315034 PMC6831096

[88] Satou Y Yasunaga J Zhao T Yoshida M Miyazato P Takai K HTLV-1 bZIP factor induces T-cell lymphoma and systemic inflammation in vivo. *PLoS Pathog.* (2011) 7:e1001274. 10.1371/journal.ppat.1001274 21347344 PMC3037353

[89] Yamamoto-Taguchi N Satou Y Miyazato P Ohshima K Nakagawa M Katagiri K HTLV-1 bZIP factor induces inflammation through labile Foxp3 expression. *PLoS Pathog.* (2013) 9:e1003630. 10.1371/journal.ppat.1003630 24068936 PMC3777874

[90] Mitagami Y Yasunaga J Kinosada H Ohshima K Matsuoka M. Interferon-gamma promotes inflammation and development of T-cell lymphoma in HTLV-1 bZIP factor transgenic mice. *PLoS Pathog.* (2015) 11:e1005120. 10.1371/journal.ppat.1005120 26296091 PMC4546626

[91] Higuchi Y Yasunaga JI Mitagami Y Tsukamoto H Nakashima K Ohshima K HTLV-1 induces T cell malignancy and inflammation by viral antisense factor-mediated modulation of the cytokine signaling. *Proc Natl Acad Sci USA.* (2020) 117:13740–9. 10.1073/pnas.1922884117 32471947 PMC7306771

[92] Sawada L Nagano Y Hasegawa A Kanai H Nogami K Ito S IL-10-mediated signals act as a switch for lymphoproliferation in Human T-cell leukemia virus type-1 infection by activating the STAT3 and IRF4 pathways. *PLoS Pathog.* (2017) 13:e1006597. 10.1371/journal.ppat.1006597 28910419 PMC5614654

